# Cortisol levels at baseline and under stress in adolescent males with attention-deficit hyperactivity disorder, with or without comorbid conduct disorder

**DOI:** 10.1016/j.psychres.2016.05.052

**Published:** 2016-08-30

**Authors:** Clare Northover, Anita Thapar, Kate Langley, Graeme Fairchild, Stephanie H.M. van Goozen

**Affiliations:** a School of Psychology, Cardiff University, UK; b MRC Centre for Neuropsychiatric Genetics and Genomics, Cardiff University, UK; c Academic Unit of Psychology, Southampton University, UK

**Keywords:** Stress, Cortisol, ADHD, Conduct disorder, Callous-unemotional traits

## Abstract

Reported findings on cortisol reactivity to stress in young people with ADHD are very variable. This inconsistency may be explained by high rates of comorbidity with Conduct Disorder (CD). The present study examined cortisol responses to a psychosocial stressor in a large sample of adolescent males with ADHD (*n*=202), with or without a comorbid diagnosis of Conduct Disorder (CD). Associations between stress reactivity and callous-unemotional traits and internalizing symptoms were also assessed. The ADHD only (n=95) and ADHD+CD (n=107) groups did not differ in baseline cortisol, but the ADHD+CD group showed significantly reduced cortisol stress reactivity relative to the ADHD only group. Regression analyses indicated that ADHD symptom severity predicted reduced baseline cortisol, whereas CD symptom severity predicted increased baseline cortisol (ADHD *β*=−0.24, CD *β*=0.16*, R*=0.26*)* and reduced cortisol stress reactivity (*β*=−0.17, *R*=0.17). Callous-unemotional traits and internalizing symptoms were not significantly related to baseline or stress-induced cortisol. Impaired cortisol reactivity is hypothesised to reflect fearlessness and is associated with deficient emotion regulation and inhibition of aggressive and antisocial behaviour. Consequently, it may partly explain the greater severity of problems seen in those with comorbid ADHD and CD.

## Highlights

• Cortisol stress responses were studied in 202 adolescent males with ADHD.
• Males with comorbid CD showed reduced cortisol stress reactivity.
• ADHD symptom severity predicted reduced baseline cortisol.
• CD symptom severity predicted increased baseline cortisol and reduced cortisol stress reactivity.
• Callous-unemotional traits and internalizing symptoms were not related to cortisol.

## 1 Introduction

The Hypothalamic-Pituitary-Adrenal (HPA) axis plays a critical role in mediating physiological responses to stress, enabling organisms to adapt to environmental changes (Marquez et al., 2006). The regulation of the HPA axis, with cortisol as its end product, appears to be dysfunctional in several psychiatric disorders (Tsigos and Chrousos, 2002). Research interest in HPA axis activity in Attention-Deficit/Hyperactivity Disorder (ADHD) has focused on the theoretical notion of under-arousal and the putative need in those with ADHD to increase their levels of arousal to avoid boredom (Zuckerman, 1994, Stadler et al., 2011). Reduced baseline cortisol levels or a blunted cortisol response to psychological stress have been found in children with ADHD compared with healthy controls (Blomqvist et al., 2007, Ma et al., 2011, Isaksson et al., 2012). However, other studies found positive associations between ADHD symptoms and cortisol in population-based samples (e.g., Palma et al., 2012) or comparable cortisol levels in children with and without ADHD (e.g., Snoek et al., 2004, Cakaloz et al., 2005, Freitag et al., 2009, Palma et al., 2012). These mixed results could be due to variations within ADHD samples, especially in relation to comorbid disorders, sample size and hormone measurement techniques (see Fairchild (2012), for a review).

Adolescents with ADHD are a heterogeneous population, with 30−50% of children with ADHD in clinical settings also meeting criteria for Conduct Disorder (CD; Biederman et al., 1991). There is clear and much more consistent evidence that HPA axis activity is altered in those with CD and Oppositional Defiant Disorder (ODD; Van Goozen et al., 2000, Kariyawasam et al., 2002, Oosterlaan et al., 2005, Van Goozen et al., 2007, Fairchild et al., 2008). It has been hypothesised that blunted cortisol reactivity reflects fearlessness and is associated with deficient emotion regulation and inhibition of antisocial behaviour (Van Goozen et al., 2007). However, previous studies on cortisol secretion in children with ADHD have not always controlled for comorbid disruptive behaviour disorders (DBDs) such as CD or ODD (e.g., Blomqvist et al., 2007). Consequently, the first aim of this study was to investigate adolescent boys with ADHD and compare those with or without a comorbid diagnosis of CD in terms of baseline cortisol and cortisol stress reactivity.

Studies that did assess and control for comorbid DBDs have still obtained mixed results. Some studies found lower baseline cortisol levels in children with ADHD and comorbid ODD/CD, but not in children with non-comorbid ADHD (Cakaloz et al., 2005, Freitag et al., 2009), whereas others found reduced baseline cortisol levels in non-comorbid ADHD (Van West et al., 2009, Ma et al., 2011) and one study found no effect of DBD comorbidity within an ADHD sample (Isaksson et al., 2012).

This may be due to differences between studies in cortisol measurement techniques or saliva collection protocols. The HPA axis is a dynamic system that not only responds to psychological and physical stress, but also exhibits a marked diurnal rhythm (Kirschbaum and Hellhammer, 1989). Therefore studies that have only assessed cortisol at one (e.g., Ma et al., 2011) or two (Cakaloz et al., 2005) time point may be difficult to interpret, especially if they have not controlled for time of awakening or time of sample collection. Furthermore, some research has relied on participants collecting cortisol samples themselves (e.g., Freitag et al., 2009, Isaksson et al., 2012), which requires participants keeping to a strict timescale and carefully following collection protocols. Problems with adherence to a saliva collection protocol might be particularly pronounced in young people with ADHD who have difficulties with concentration, organisation and being forgetful; this could lead to both false positive and false negative findings (Kudielka et al., 2004).

Even when cortisol reactivity to a stressor has been investigated, there are inconsistent findings. Reduced cortisol reactivity to stress has been found in children with ADHD and comorbid DBD compared to children with ADHD alone (Hastings et al., 2009, Snoek et al., 2004). Other studies found associations between ADHD symptoms and reduced cortisol stress reactivity after controlling for comorbid DBD (Van West et al., 2009, Pesonen et al., 2011). However, the choice of stressor is important. For example, some previous studies have used inadequate or relatively weak stressors, such as cognitive tests (e.g., Yang et al., 2007). The present study used an established psychosocial stress induction protocol that elicited feelings of anger, failure and negative social evaluation (e.g., Van Goozen et al., 2000, Snoek et al., 2004, Fairchild et al., 2008), and involved collecting eight cortisol samples under strict experimental conditions.

When analysing the effects of CD, it is also important to assess the effects of anxiety/depression symptoms as it is increasingly recognised that comorbidity between externalizing and internalizing problems is common (Lahey and Waldman, 2012). Anxiety or depressive symptoms are frequently reported to be associated with *increased* cortisol activity or reactivity (Knight et al., 2010). Thus patterns of cortisol reactivity in ADHD can be further complicated by patterns of comorbid emotional symptoms as well as conduct disorder (e.g., Hastings et al., 2009).

Another potentially important source of heterogeneity that is closely related to conduct disorder, is variation in callous-unemotional (CU) traits. These traits identify those at greater risk for severe antisocial behaviour (Lahey and Waldman, 2012) and reduced responsiveness to treatment (Hawes et al., 2014). The importance of such traits has been acknowledged by including limited prosocial emotions as a specifier for CD in the fifth edition of the Diagnostic and Statistical Manual of Mental Disorders (American Psychiatric Association, 2013). CU traits have been linked to lower baseline cortisol levels and a blunted cortisol response to stress (O’Leary et al., 2007). However, the impact of CU traits on cortisol activity has been predominantly investigated in non-clinical samples free of psychiatric disorders (Loney et al., 2006). An exception to this was a study that reported reduced cortisol responses to stress in participants with ADHD and high levels of CU traits – with over half of the participants having a comorbid DBD diagnosis – compared to those with ADHD with low levels of CU traits (Stadler et al., 2011). This finding now needs to be replicated in a larger clinical sample of adolescents with ADHD (the sample size in the latter study was N=36).

Previous research has also focused on heterogeneity in cortisol reactivity between ADHD subtypes. Maldonado et al. (2009) observed reduced overall cortisol levels in hyperactive/impulsive children compared with inattentive ADHD children during a psychosocial stress induction procedure. However, their stress procedure was conducted in the morning and failed to induce an increase in cortisol. Van West et al. (2009) reported blunted cortisol responses in combined ADHD children when compared with a group of inattentive children. However, this group also had higher DBD symptoms. Hastings et al. (2009) controlled for comorbid disorders and found that ADHD subtypes were not differentially associated with baseline or reactive cortisol levels. However, comorbid DBD predicted decreased cortisol reactivity in boys with inattentive and hyperactive subtypes of ADHD, but not in boys with combined subtype of ADHD. This study did not focus on ADHD subtype classifications because previous publications (e.g. Willcutt et al., 2012) have demonstrated that these subtypes are not definitive or stable over time. Instead we examined the role of symptom severity within each dimension on cortisol.

The present study aimed to assess cortisol levels at baseline (pre-stress samples taken under experimental conditions) and in response to stress (area under curve with respect to increase; Pruessner et al., 2003) in a sample of adolescent males with ADHD, and explore the contributions of Conduct disorder diagnosis, ADHD severity, CD symptom severity, CU traits and internalizing symptoms. To our knowledge, this is the largest study of experimentally-induced stress reactivity in an ADHD or CD sample.

## 2 Methods

### 2.1 Sample

Participants were recruited from Child and Adolescent Mental Health Services and Community Child Health Clinics in Wales. Children in the sample were males of British Caucasian origin (also being recruited for a genetics study; Van Goozen et al., 2015) and had a clinical diagnosis of ADHD. Those with any known clinical or research diagnosis of schizophrenia, bipolar disorder, Autistic Spectrum Disorder (ASD), Tourette's syndrome, or with an IQ<70, epilepsy, brain damage or any other neurological or genetic disorder were excluded from the study. In total, 202 adolescent males with ADHD (mean age=13.95 years, sd=1.82; age range 10–17 years) took part in the present study. No participants were stimulant naïve but participants who were currently being prescribed stimulant medication were asked to come off their medication at least 24 h prior to testing.

Ethical approval was obtained from the Wales Multicentre Research Ethics Committee. Informed written consent was obtained from all parents and adolescents aged over 16 years whereas written assent was obtained from adolescents below age 16 years.

### 2.2 Clinical measures

Child psychopathology was assessed using the Development and Well Being Assessment (DAWBA) structured psychiatric research diagnostic interview using both parents and children as informants (Goodman et al., 2000). This interview has been used widely to assess child and adolescent psychopathology in clinical and large population studies and has shown to be a reliable diagnostic interview. The interview involves assessment of all symptoms and criteria necessary to generate DSM-IV and ICD-10 diagnoses. Parents completed the ADHD and ODD/CD sections and children the ODD/CD section of the DAWBA. All interviews were administered by trained psychologists, supervised by an experienced clinician (AT). Symptom scores and diagnoses were generated from the DAWBA interview. Total symptom scores and diagnoses were computed according to DSM-IV criteria (the DSM-5 had not been published at the start of the study) (APA, 2000). Diagnoses were further verified by a trained clinician. CD symptoms were considered present if endorsed by either the parent or child. Parent-rated emotional/anxiety symptoms were assessed using the Strengths and Difficulties Questionnaire (SDQ; Goodman et al., 2000). This is a very extensively used questionnaire used to assess child psychopathology with high reliability and validity. The five SDQ emotional items (worries, unhappy, afraid, clingy, somatic) were scored on a 3-point Likert scale and summed to obtain a total emotional symptom score (score range 0–10).

Participants were allocated to two groups according to whether they met DSM-IV diagnostic criteria for Conduct Disorder or not: ADHD only (*n*=95) and ADHD with comorbid CD (ADHD+CD; *n*=107). In addition, 48.1% of the sample met diagnostic criteria for ODD (48.6% for those with ADHD only and 46.5% for those with ADHD+CD). However, the focus of the paper concentrated on the more severe area of conduct disorder.

Callous-Unemotional (CU) traits were measured using the Youth Psychopathic traits Inventory (YPI; Andershed et al., 2002). The CU subscale of the YPI contains 15 items, and each item is answered on a 4-point Likert scale (score range 15–60). The reliability and convergent validity of the YPI with other measures of CU traits has been established (Andershed et al., 2007).

Cognitive ability was assessed in all participants using the Wechsler Abbreviated Scale of Intelligence (WASI; Wechsler, 1999) – 2-subtest form (vocabulary and matrix reasoning).

### 2.3 Psychosocial stress induction procedure

**Fig. 1:**
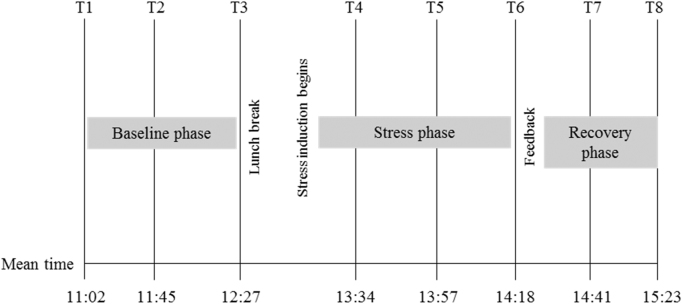
Schematic representation of the test procedure and mean cortisol and mood rating sampling times.

Participants arrived at our laboratory in the morning and completed a battery of questionnaires and neuropsychological tests including the WASI before lunch. During this time they were asked to provide three baseline cortisol samples approximately 40 min apart from each other. After lunch they were informed that they would be taking part in a competition with an opponent of a similar age with a cash prize for the winner. This procedure is described in detail elsewhere (Van Goozen et al., 2000, Fairchild et al., 2008); briefly, it involves inducing provocation and frustration between the participant and a pre-recorded video opponent who are both competing for a cash prize. The competition begins with a frustration-inducing game in which the participant performs a difficult, computer-based manual precision task under time pressure while believing they are being watched by the video opponent and experimenter. By design, all participants fail to achieve their target score and receive negative evaluations of their performance from the opponent. This feedback is standardised by using a video recording of the competitor, who criticises the participant's performance in a competitive and derogatory way. Following this task, participants complete three further challenging tasks aimed at increasing performance uncertainty and sense of failure. Three stress cortisol samples were taken during the competition, approximately 20 min apart from one another. Finally, mood was restored by the participant watching their opponent perform poorly, resulting in the participant winning the competition (and the cash prize). Two post-stress recovery samples were collected 20 min apart while participants completed some final non-challenging tasks and questionnaires. Please see Fig. 1 for a schematic representation of the stress-induction procedure.

### 2.4 Procedure for saliva collection and analysis

A synthetic swab (polyethylene) was placed into the mouth and chewed/sucked on for 60 s, after which it was placed into a plastic sample tube (Salivettes) and stored at −20 °C. Cortisol levels were determined employing a competitive solid phase time-resolved fluorescence immunoassay with fluoromeric end point detection. The intra-assay coefficient of variation was between 4.0% and 6.7%, and the corresponding inter assay coefficients of variation were between 7.1% and 9.0% (Dressendörfer et al., 1992). Results are reported in nmol/L.

### 2.5 Self-rated emotions

Participants rated their emotional responses eight times using an adaptation of a clinical self-rating scale (Von Zerssen, 1986). The scale contained 11 items (happy/gloomy, well/sick, cheerful/not cheerful, good/bad, liked/not liked, satisfied/not satisfied, worried/not worried, embarrassed/not embarrassed, ashamed/not ashamed, afraid/not afraid, and angry/not angry), which participants rated using 9-point ordinal scales. Subjective ratings occurred at the same times as saliva collection.

### 2.6 Data analyses

Two participants were excluded because of missing or incomplete DAWBA data. A cortisol baseline measure was calculated from the average of the three cortisol samples taken before the stress phase of the testing day began. The area under the curve with respect to increase (AUCi) was used to quantify cortisol reactivity, making use of the repeated measurements and emphasizing the change over time rather than the starting level (Pruessner et al., 2003). A negative mood score was calculated for each of the cortisol sampling times which was the sum of the 11 emotion ratings and the scores for positive emotions were reversed during scoring. Ten participants had taken ADHD medication within the past 24 h; however, their cortisol levels did not differ from the rest of the sample (*p*=0.42 for baseline; *p*=0.33 for AUCi), so these cases were included in the analyses. However, the main analyses (assessing between-group differences in baseline, AUCi and mood) were rerun without these participants and the results remained the same after removing them.

Between group differences were assessed using ANOVAs, with Greenhouse-Geisser correction applied where assumptions of sphericity were violated. Effect sizes are reported as eta squared (*η*^*2*^; small≥0.01, medium≥0.06, large≥0.14; Cohen, 1988). Pearson's correlations and multiple regressions examined the effect of the clinical characteristics on baseline cortisol and cortisol stress reactivity. Analyses were carried out using SPSS 20.0 (SPSS Inc., Chicago, IL).

## 2 Results

The demographic data for the two subgroups, and the results of between-group analyses are presented in Table 1.

The ADHD only and ADHD+CD groups differed significantly in terms of ADHD, ODD and CD symptom severity, as well as CU traits, but there was no difference in emotional symptoms as measured by the SDQ. Although there was a significant group difference in IQ, IQ was not significantly associated with cortisol ( $r_{baseline\;\;cortisol}=-0.11$, *p*>0.05; *r*_AUCi_=0.14, *p*>0.05). IQ was therefore not included as a covariate in subsequent between-group analyses.

### 2.1 Self-reported emotions

There was a main effect of time, *F*(3.39, 595.70)=56.71, *p*<0.001, *η*^*2*^=.24, but no effect of group [*F*(1, 176)=0.86, *p*=0.37, *η*^*2*^=0.04] and no significant group×time interaction [*F*(3.39, 595.70)=2.07, *p*=0.10, *η*^*2*^=0.01], indicating that the stress paradigm induced negative emotions in both groups to an equal extent (see Fig. 1).

### 2.2 Cortisol

The ADHD and ADHD+CD groups did not differ in baseline cortisol *F*(1, 201)=2.24, *p*=0.14, *η*^*2*^=0.01). There was a main effect of time [*F*(3.59, 689.04)=49.88, *p*<0.001, *η*^*2*^=0.21] but no effect of group [*F*(1,192)=0.05, *p*=0.82, *η*^*2*^=0.00] and no group×time interaction [F(3.59, 689.04)=1.71, p=0.15, *η*^*2*^=0.01]. However, analysis of the more sensitive AUCi values for the cortisol response showed a significant group difference, [*F*(1, 201)=6.33, *p*=0.01, *η*^*2*^=0.04], reflecting more pronounced cortisol stress reactivity in the ADHD than the ADHD+CD group.

**Fig. 2:**
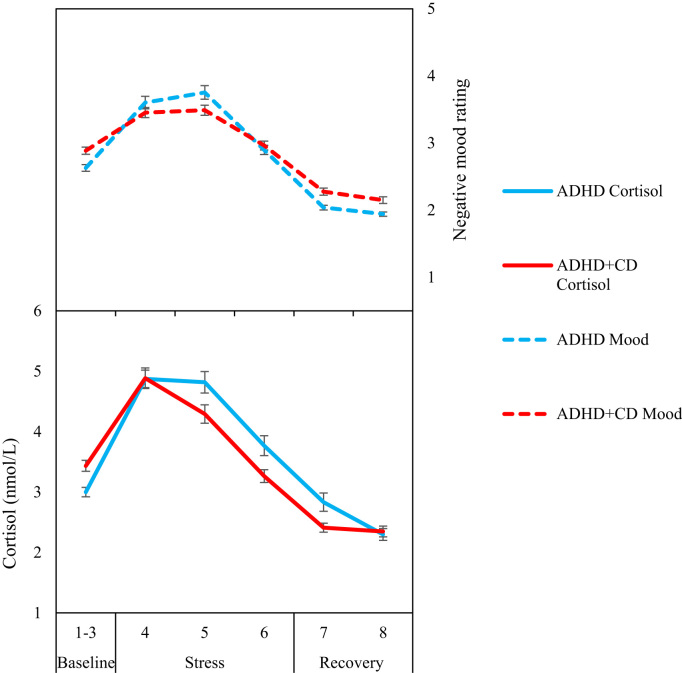
Mean cortisol levels and negative mood scores during baseline, stress and recovery phases for the ADHD and ADHD+CD groups. Error bars show±1 standard error.

Fig. 2 presents schematically the cortisol and negative mood profiles of ADHD and ADHD+CD groups, illustrating that the groups had similar self-reported mood profiles, but different cortisol profiles with the ADHD+CD group showing an attenuated cortisol response to stress.

Table 2 shows that ADHD (both hyper-impulsive and inattentive), ODD and CD symptoms were all significantly correlated with baseline cortisol. However, only ADHD and CD symptom severity significantly predicted variance in baseline cortisol in a regression analysis (*F*=6.88, *p*=0.001, *R*=0.26). ADHD symptoms were inversely related to baseline cortisol, whereas CD symptoms were positively related to baseline cortisol (see Table 3). Anxiety scores and CU traits did not correlate with cortisol levels.

Only CD symptoms showed a significant inverse correlation with cortisol reactivity (AUCi); a regression showed that CD severity significantly predicted cortisol reactivity (*F*=6.19, *p*=0.01, *R*=0.17; see Table 4).

## 3 Discussion

The aim of this study was to investigate whether with a sample of those with ADHD, a pattern of reduced HPA activity was specific to those with ADHD and comorbid CD relative to those with ADHD alone by investigating both cortisol baseline and stress reactivity levels. The second aim was to analyse dimensionally the effect of ADHD and CD symptom severity, CU traits and emotional symptoms on cortisol baseline levels and stress reactivity. To this end, we studied cortisol levels in 202 male adolescents with ADHD, of whom 107 also met criteria for a diagnosis of CD, under baseline conditions and during a psychosocial stressor that involved frustration and competition. No differences were found between the two groups (ADHD vs. ADHD+CD) in baseline cortisol levels. These results are in contrast with some studies reporting lower baseline cortisol levels in children with ADHD and comorbid DBD compared to children with ADHD only (Cakaloz et al., 2005), but are in line with other findings (Snoek et al., 2004).

When looking at the variables continuously, however, both ADHD and CD symptom levels predicted baseline cortisol. CD symptoms positively related to baseline cortisol levels, whereas ADHD symptom severity inversely predicted baseline cortisol. Imeraj et al. (2012) found a flatter cortisol slope in their ADHD only group, which was explained by morning hypo-arousal and evening hyper-arousal, whereas the ADHD+ODD group showed a steeper cortisol slope with morning hyper-arousal and evening hypo-arousal. We have previously found a trend towards higher baseline cortisol levels when measuring cortisol during the day in CD adolescents (Fairchild et al., 2008), which is in agreement with the positive correlation cortisol levels in found for CD symptoms. We found that both inattentive and hyperactive-impulsive symptoms inversely correlated with cortisol baseline, although hyperactive-impulsive symptoms were more strongly correlated. This supports previous studies (e.g. Maldonado et al., 2009) that found reduced cortisol levels in children displaying the hyperactive-impulsive presentation of ADHD compared to those displaying the inattentive presentation.

In terms of cortisol stress reactivity, the findings are consistent with previous results showing reduced cortisol stress responses in adolescent males with comorbid ADHD and ODD when compared to those with ADHD alone (Snoek et al., 2004). The current findings showed significantly reduced reactivity – as measured by the area under the curve with respect to increase (AUCi) - in those with ADHD+CD compared to those with ADHD only. Interestingly, the two diagnostic groups did not differ in self-reported negative emotions, with both groups reporting an increase in negative moods during stress exposure. Thus we observed in the ADHD+CD group a larger discrepancy between the intensity of self-reported negative moods and cortisol reactivity, whereas the non-comorbid ADHD group showed parallel increases in negative moods and cortisol.

We found that CD severity was the only predictor of our cortisol reactivity measure (AUCi); although the CD group had more ADHD symptoms, these did not significantly correlate with the AUCi measure. CU traits did not predict cortisol levels at baseline or stress reactivity. ODD symptoms were inversely correlated with baseline cortisol, but they did not enter the model with ADHD and CD symptoms when using a stepwise regression. The lack of an association between cortisol and CU traits contradicts previous research that found reduced cortisol stress reactivity in ADHD participants with high CU traits (Stadler et al., 2011), but a possible explanation for this discrepancy is that the ADHD and high CU traits group were also higher in conduct problems than the low CU traits group. Our study suggests that CD symptom severity is more important in explaining differences in cortisol reactivity than variation in CU traits. Few studies have looked at clinical measures continuously and some inconsistent findings could therefore have been caused by the varying methods used for eliciting a cortisol response.

It is important to note that CD was not associated with hypo-activity of the HPA axis at baseline, but rather a specific *hypo-reactivity* during stress. This has implications for arousal-based theories of CD behaviour (Van Goozen et al., 2007). The absence of lower baseline cortisol levels in CD is incompatible with the hypothesis that CD children are driven to their behaviour by stimulation-seeking motives (Zuckerman, 1994); instead, reduced reactivity findings are more supportive of fearlessness accounts (Van Goozen et al., 2007). A possible explanation is that more frequent exposure to stress may result in adrenocortical habituation among CD children to some types of stress, leading to reduced stress reactivity. However, the apparently diminished reactivity of the HPA axis in CD children was not related to their perception and interpretation of the stressor; the ADHD and ADHD+CD groups perceived the stressor as equally threatening and frustrating. It is possible that HPA axis reactivity and subjective arousal are less well coordinated in children with CD, perhaps due to the effects of stressful events in early life that could partly be evoked by ADHD behaviours (Harold et al., 2013). Early life stress could lead to alterations in developing neurobiological systems including the HPA axis (Lupien et al., 2009) as could genetic factors (Bartels et al., 2003).

The results of this study indicate that amongst adolescent boys with ADHD, a pattern of reduced HPA reactivity during stress is observed in those with comorbid CD. The better prognosis of ADHD relative to ADHD+CD could reflect the results of intact responsivity to social conditioning due to increased reactivity of the HPA axis or due to greater exposure to stressors leading to both increased aggression and HPA axis dysfunction in the comorbid group. It is also relevant from a clinical point of view that we found that the pattern of low HPA axis activity under stress was more apparent in those with more severe conduct problems. If HPA axis dysfunction is a key risk mechanism mediating the developmental pathway from ADHD to CD, modulating this system may be helpful in the treatment of children with ADHD - either through psychosocial and/or pharmacological interventions (Stadler et al., 2011, Van Goozen et al., 2007). A better understanding of the mechanisms involved in the development, persistence and prognosis of disruptive behaviour disorders should ultimately result in more effective interventions.

### 3.1 Strengths and limitations

This was the largest study to date to investigate stress reactivity in adolescents with a clinical diagnosis of ADHD. The participants were well-characterized from a psychiatric perspective with information collected from multiple informants. We collected multiple saliva samples under highly controlled experimental conditions, and used an effective psychosocial stressor to induce cortisol reactivity, as well as measuring subjective responses to the stressor. However, the present study was also subject to a number of limitations. We did not include a healthy control group or a ‘pure’ CD group for comparison with the ADHD or ADHD+CD groups. However, in previous studies using the same paradigm, we assessed such groups and were able to show that healthy controls show a significant cortisol stress responses, whereas this reaction is blunted or absent in children or adolescents with CD or ODD (Van Goozen et al., 2000, Snoek et al., 2004, Fairchild et al., 2008). Moreover, we have also demonstrated that boys with ADHD alone (i.e., without CD) show a similar stress response to healthy controls (Snoek et al., 2004). The aim of the current study was to supersede previous group-based comparisons and to examine the role CD and other symptoms in cortisol stress reactions within those with ADHD.

We did not systematically collect information on exposure to significant early life events and can therefore only speculate about the possible mechanisms underlying our findings. Children with CD have been exposed to significantly greater environmental adversity than children with ADHD alone (Schachar and Tannock, 1995), and the reduced HPA axis reactivity that we found in the ADHD+CD group could therefore have been caused by differences in the early lives of the children we studied (Isaksson et al., 2013). Longitudinal prospective studies from an early age onwards are needed in order to investigate the effect of adverse early life events on HPA axis (re)activity in a more detailed way. Genetic factors are also known to contribute strongly to the presence of conduct problems in ADHD (Thapar et al., 2007) as well as stress responsiveness (Wüst et al., 2000). Thus the contribution of genetic risk factors will also need to be examined in future investigations.

We found no effect of anxiety and depressive symptoms on cortisol baseline or reactivity in contrast to other studies (e.g., Pruessner et al., 2003). Although we used a widely used, reliable and valid questionnaire (the SDQ) to measure internalizing symptoms, the internalizing subscale only contains five items and there may not have been sufficient variability within the sample to detect any significant effects.

This study was not designed to take into account the effects of diurnal variation in cortisol secretion. We collected eight samples spread over an experimental testing day (approximately five hours) and the stress phase always occurred in the afternoon, which is the part of the day that is least affected by varying circadian rhythms (Maheu et al., 2005).

We had no ethical permission, nor did we want to insist that participants should come off their medication for a longer period of time; it could have put more severe cases off from taking part in the research. Therefore, although we controlled for the acute side effects of the medication, we could not control the longer term effects of drug use in this study. Further research, that is able to analyse the longer term effects of ADHD medication on stress reactivity is needed.

Finally, although the present study shows that males with ADHD and CD show a weaker neuroendocrine response to challenges that involve frustration and provocation than those with ADHD alone, it is not clear to what extent these findings would generalize to other types of stress (although see Popma et al., 2006). Future research should examine reactivity to fear-inducing challenges.

In conclusion, this study of adolescent boys with ADHD found a significant difference in cortisol reactivity to a psychosocial stressor between participants with ADHD alone and those with ADHD and comorbid CD. Impaired cortisol reactivity is hypothesised to reflect fearlessness and has been associated with deficient emotion regulation and inhibition of aggressive and antisocial behaviour. Consequently, it may partly explain the greater severity of behavioural problems seen in those with comorbid ADHD and CD. Future research should use longitudinal research designs to study the effects of early adversity on the development of externalizing problems and HPA axis functioning in order to identify more optimal strategies for timely intervention.

## Author contributions

Conceived and designed the experiment: SHMvG, AT, KL, GF. Performed the experiments: CN. Analyzed the data: CN, SHMvG. Wrote the paper: CN, SHMvG, AT, KL, GF.

## Disclosure of conflicts of interest

CN, AT, KL, GF and SHMvG report no conflicts of interest.

## Figures and Tables

**Table 1:** Means (with SDs) for the demographic and clinical characteristics of the ADHD and ADHD+CD subgroups.

	*ADHD*	*ADHD+CD*	
*(n=95)*	*(n=107)*	*P value*
Age	13.74 (1.87)	14.10 (1.76)	n.s.
IQ	90.23 (10.24)	84.31 (9.35)	p<0.001
ADHD	11.74 (4.91)	13.15 (4.17)	p<0.05
CD	0.96 (.78)	5.69 (2.41)	p<0.001
ODD	2.84 (2.46)	4.73 (2.65)	p<0.001
CU traits	31.38 (6.01)	35.64 (6.99)	p<0.001
SDQ-Emotional	4.81 (2.63)	4.96 (2.37)	n.s.

*Note:* All between group analyses were done using independent samples t tests; ADHD=number of ADHD symptoms; CD=number of CD symptoms; ODD=number of ODD symptoms; CU traits=callous-unemotional traits subscale score; SDQ-Emotional=Strengths and Difficulties emotional symptom subscale score.

**Table 2:** Pearson's correlations of clinical characteristics and different measures of cortisol secretion.

	ADHD	Hyper-Impulsive	Inattentive	CD	ODD	CU traits	SDQ-Emotion	Baseline cortisol	AUCi cortisol
ADHD	–								
Hyper-Impulsive	0.91**	–							
Inattentive	0.87**	0.59**	–						
CD	0.19**	0.29**	0.08	–					
ODD	0.57**	0.58**	0.43**	0.42**	–				
CU traits	0.11	0.07	0.13	0.40**	0.18*	–			
SDQ-Emotion	0.32**	0.33**	0.22**	0.10	0.32**	−0.07	–		
Baseline cortisol	−0.20**	−0.20**	−0.15*	0.14*	−0.14*	−0.05	−0.05	–	
AUCi cortisol	−0.05	−0.04	−0.05	−0.17*	0.06	−0.11	0.08	−0.36**	–

*Note:* ADHD=number of ADHD symptoms; Hyper-Impulsive=number of hyperactive and impulsive ADHD symptoms; Inattentive=number of inattentive ADHD symptoms; CD=number of CD symptoms; ODD=number of ODD symptoms; CU traits=callous-unemotional traits subscale score; SDQ-Emotional=Strengths and Difficulties emotional symptom subscale score; Baseline cortisol=average of three baseline cortisol samples; AUCi cortisol=area under the curve with respect to increase.

* p<0.05.

** p<0.001.

**Table 3:** Regressions of clinical predictors on baseline cortisol.

Step		*b*	*SE b*	*β*

1	(Constant)	4.04	0.34	
	ADHD symptoms	−0.07	0.03	−0.21**

2	(Constant)	3.87	0.34	
	ADHD symptoms	−0.09	0.03	−0.24**
	CD symptoms	0.09	0.04	0.16*

*Note: R*^*2*^*for step 1=0.04;* Δ*R*^*2*^*for step 2=0.03.*

* p<0.05.

** p<0.001.

**Table 4:** Regressions of clinical predictor on cortisol stress reactivity.

Step		*b*	*SE b*	*β*
1	(Constant)	16.68	49.54	
	CD symptoms	−26.94	10.82	−0.17*

*Note: R*^*2*^*for step 1=0.03*

* p<0.05
